# Reality shock and mental resilience among adolescent police cadets: meaning, identity, and relational pathways in a Chinese police academy context

**DOI:** 10.3389/fpsyt.2026.1921313

**Published:** 2026-10-05

**Authors:** Hongping Lin

**Affiliations:** Jiangsu Police Institute, Nanjing, China

**Keywords:** adolescent police cadets, Chinese police academy, identity work support, mental resilience, occupational calling, reality shock, relational job crafting

## Abstract

**Introduction:**

Adolescent police cadets are trained in an institutional environment where schooling, collective discipline, physical training, peer living, and early occupational socialization are closely intertwined. This environment may provide structure and purpose, but may also expose cadets to a distinctive form of vocational environmental risk. However, the psychological pathways through which environmental risk and personal resources are linked to mental resilience in this population remain underexplored.

**Methods:**

Drawing on an environmental risk and resilience framework, this cross-sectional study examined the association between reality shock and mental resilience among 642 adolescent police cadets from three police education institutions in China, and explored whether positive psychological traits were linked to resilience through occupational calling, identity work support, and relational job crafting. The sample was randomly split into an exploratory subsample (n = 321) and a confirmatory subsample (n = 321), resulting in a refined 36-item measurement model. Structural equation modeling and bootstrap mediation analyses (5,000 resamples) were conducted on the full sample.

**Results:**

Reality shock was negatively associated with mental resilience (β = -.23, p < .001), whereas positive psychological traits, occupational calling, identity work support, and relational job crafting were positively associated with resilience. Competing nested structural models and directional alternative model tests suggested that a combined parallel-sequential mediation model best fit the data. Bootstrap analyses confirmed that the indirect effects through the three parallel pathways and the sequential pathway were all significant. Multi-group analyses across academic years supported strict measurement invariance and suggested broadly similar structural associations across training stages.

**Discussion:**

These findings suggest that resilience in adolescent police cadets is not merely individual toughness; it is deeply linked to occupational meaning, identity support, and relational practices. The results extend environmental risk and resilience frameworks to specialized vocational education settings and offer practical implications for police education programs. Because the data were cross-sectional and self-reported, causal conclusions cannot be drawn.

## 1 Introduction

Adolescence is a developmental period in which mental health is highly responsive to social and institutional environments. Young people are still developing emotional regulation, identity coherence, peer belonging, future orientation, and strategies for coping with pressure. For this reason, adolescent mental health cannot be explained only by individual dispositions. It is shaped through continuous interaction with families, schools, peers, communities, digital spaces, and broader social systems (1–4). Environmental risk and resilience perspectives further suggest that risk and protection often operate together rather than separately (5–8).

Existing research on adolescent environmental risk has mainly examined family adversity, school climate, academic pressure, peer victimization, neighborhood disadvantage, and online exposure. These lines of research are important, but they do not fully capture specialized vocational education settings in which adolescents live under institutional rules that are already shaped by a future occupation. Police academies represent such a setting. Adolescent police cadets are students, but their daily routines are organized around becoming future police officers. They study law and procedure, participate in physical training, follow collective schedules, live in dormitories, practice command and cooperation, and receive repeated messages about duty, discipline, obedience, restraint, courage, and public service.

Police academy life therefore differs from ordinary schooling. It contains academic requirements, but it also includes early occupational socialization, quasi-organizational discipline, hierarchical communication, collective management, and role expectations associated with law enforcement. These features may provide protective structure, belonging, and purpose. At the same time, they may expose cadets to pressures that cannot be reduced to ordinary academic stress. Cadets may discover that police work is less simple, less heroic, more routine, more constrained, and more emotionally demanding than they imagined before admission. This expectation-reality discrepancy is central to the present study.

Reality shock refers to discomfort, confusion, or disillusionment that occurs when individuals encounter a mismatch between prior expectations and the actual experience of a role, organization, or occupation (9–12). Previous research has usually examined reality shock in organizational entry, early employment, or work role transition. However, for police cadets, reality shock may appear before formal employment. Many cadets enter police education with images of policing shaped by media, family expectations, entrance publicity, public narratives, and personal ideals. Training gradually complicates these images through repetitive drills, procedural requirements, emotional restraint, physical fatigue, hierarchical discipline, peer comparison, and instructors’ accounts of occupational stress.

In the police academy context, reality shock should be distinguished from general academic setback. Academic setback usually refers to poor grades, examination stress, or difficulty mastering school subjects. Police academy reality shock concerns a broader mismatch between cadets’ imagined police role and the institutional, relational, ethical, and embodied realities of police training. It may include task-related shock, such as repetitive drills and demanding physical training; organizational shock, such as hierarchical discipline and limited autonomy; emotional shock, such as the expectation of restraint under stress; relational shock, such as peer comparison and command-based communication; and occupational-ethical shock, such as recognizing the routine, constrained, or ambiguous nature of police work. These subtypes clarify why police academy reality shock is not identical to ordinary student disappointment.

This study treats reality shock as a vocational environmental risk factor. It is vocational because the shock concerns the anticipated world of police work. It is environmental because the shock emerges through training routines, organizational messages, peer conversations, instructor interactions, field observations, and broader cultural meanings of policing. For adolescent cadets, whose identity and future orientation are still developing, this form of risk may be especially important.

The outcome of interest is mental resilience. Resilience refers to the capacity to maintain or regain adaptive functioning under adversity, stress, or developmental challenge (13–16). In police education, resilience should not be reduced to silent endurance or unquestioning toughness. A resilient cadet is not someone who never feels tired, confused, or disappointed. Rather, resilience may involve recovering after poor performance, adjusting after criticism, seeking support when needed, staying connected with personal values, and integrating difficult experiences into a more realistic understanding of future police work. Developmental resilience research emphasizes that resilience is not located only inside the individual but depends on the interaction between personal resources and environmental opportunities (5–7).

Positive psychological traits may serve as one set of personal resources. These traits include optimism, hope, self-efficacy, perseverance, and a general tendency to approach difficulties as manageable rather than overwhelming (17–21). Among adolescent police cadets, such traits may be associated with stronger resilience because they support adaptive interpretation and coping during demanding training. However, positive psychological traits do not operate in an empty space. Their association with resilience may depend on whether cadets can connect personal strengths with occupational meaning, identity work support, and constructive relationships.

Occupational calling is one meaning-based pathway. Calling refers to the experience of work as purposeful, socially valuable, and connected with one’s sense of self (22–25). Among police cadets, calling may appear as the belief that police work contributes to safety, order, fairness, and public service. Calling may help cadets interpret hardship as meaningful rather than merely burdensome. Yet calling may also be fragile if it depends only on heroic or idealized images of policing. A more mature calling includes both purpose and realism.

Identity work support is a second pathway. Identity work refers to the effort people make to construct, revise, defend, or express who they are in relation to social roles and institutional expectations (26–29). Adolescence is already a period of identity exploration. Police education adds a professional identity task: cadets are expected to become police officers. This role includes tensions between authority and service, discipline and discretion, emotional control and empathy, organizational loyalty and legal responsibility. If instructors, peers, mentors, and institutional routines provide support for identity reflection, cadets may be more able to integrate confusion into development rather than conceal it as weakness.

Relational job crafting is a third pathway. Job crafting refers to proactive changes individuals make in the task, cognitive, or relational boundaries of their work (30–32). Although adolescent cadets are not yet full police employees, academy life contains work-like elements: group training, command structures, evaluation, simulation, field-related observation, and collective task completion. Relational job crafting in this context may include seeking advice from instructors, building trust with classmates, learning from senior cadets, joining study or training groups, and approaching mentors after difficult experiences. In a highly structured academy environment where students cannot easily change formal rules, relationships may be one of the few spaces through which cadets actively shape their adaptation.

Crucially, these three pathways can be structured in a sequential order. Occupational calling provides a broad motivational foundation, which can drive the clarification of a professional self (identity work). Once a cadet feels secure in their professional identity, they may be more likely to engage proactively with their environment (relational job crafting). Therefore, while parallel mechanisms are expected to carry the primary associations, a sequential mechanism (Calling → Identity → Crafting) provides a theoretically coherent supplementary framework for understanding how broad occupational ideals translate into proactive coping behaviors.

Despite related work on adolescent resilience, police occupational stress, calling, identity, and job crafting, several gaps remain. First, adolescent police cadets have rarely been examined as a distinct developmental group situated between ordinary schooling and occupational socialization. Second, police stress research has focused mainly on serving officers or adult recruits, leaving limited evidence on expectation-reality discrepancies during police academy education. Third, reality shock has often been treated as a general transition experience, but its police academy-specific dimensions have not been clearly differentiated from ordinary academic setbacks. Fourth, calling, identity work support, and relational job crafting have usually been examined separately, although they may operate interactively in collective and disciplined police academy environments. Fifth, adapted job crafting measures have rarely been validated specifically for adolescent police cadets, leaving uncertainty about whether relational job crafting can be measured reliably in this context.

Based on the above reasoning, the following hypotheses were proposed.

 Hypothesis 1. Reality shock will be negatively associated with mental resilience among adolescent police cadets.
 Hypothesis 2. Positive psychological traits will be positively associated with mental resilience among adolescent police cadets.
 Hypothesis 3. Occupational calling will mediate the association between positive psychological traits and mental resilience.
 Hypothesis 4. Identity work support will mediate the association between positive psychological traits and mental resilience.
 Hypothesis 5. Relational job crafting will mediate the association between positive psychological traits and mental resilience.
 Hypothesis 6. Occupational calling, identity work support, and relational job crafting will operate as parallel mediators in the association between positive psychological traits and mental resilience.
 Hypothesis 7. A theoretically plausible sequential mediation pathway will also be examined, in which positive psychological traits are linked to stronger occupational calling, stronger occupational calling is linked to greater identity work support, greater identity work support is linked to more relational job crafting, and relational job crafting is linked to higher mental resilience.

## 2 Materials and methods

### 2.1 Participants and procedure

A cross-sectional questionnaire survey was conducted among adolescent police cadets from three police education institutions in China. The institutions were selected because they enrolled cadets in late adolescence and provided a combination of academic instruction, disciplinary management, physical training, collective living, and practice-oriented police education. Data were collected during the spring semester of 2026. Before data collection, the research team contacted student affairs offices and course instructors to explain the research purpose and the voluntary nature of participation.

Participants completed the questionnaire either in classroom settings or through a secure online questionnaire platform arranged by the institution. The survey was anonymous. Cadets were told that the study was not part of course assessment, police training evaluation, or administrative review. They were also informed that they could stop answering at any time. No names, student numbers, phone numbers, or other direct identifiers were collected. The first page of the questionnaire presented the informed consent statement. Only cadets who confirmed consent could continue.

A total of 681 questionnaires were returned. After excluding responses with substantial missing data, repeated response patterns, or unrealistically short completion times, 642 valid questionnaires remained. The valid response rate was 94.3 percent. Participants ranged from 16 to 20 years old, with a mean age of 18.27 years and a standard deviation of 1.08. The sample included 407 male cadets and 235 female cadets. There were 218 first-year cadets, 207 second-year cadets, and 217 third-year cadets. **Table 1** presents the demographic characteristics of the sample.

**Table 1:** Demographic characteristics of adolescent police cadets.

Variable	n	%
Gender		
Male	407	63.4
Female	235	36.6
Academic year		
First year	218	34.0
Second year	207	32.2
Third year	217	33.8
Family residence		
Urban area	261	40.7
County or township	239	37.2
Rural area	142	22.1
Only-child status		
Only child	294	45.8
Not only child	348	54.2
Close family member with police experience		
Yes	126	19.6
No	516	80.4

N = 642. Participants were 16 to 20 years old.

### 2.2 Translation, adaptation, and item selection

Because the scales were adapted to the police academy context, rigorous translation and psychometric procedures were implemented. Existing Chinese versions were used when available. For scales originally in English, a back-translation procedure was used. Two bilingual researchers independently translated the items into Chinese, and a third bilingual researcher back-translated them into English to ensure semantic equivalence.

An initial pool of 56 items was generated. Five experts with backgrounds in adolescent psychology, police education, organizational behavior, and psychological measurement evaluated each item for relevance, clarity, and contextual appropriateness. A pilot test with 36 adolescent police cadets was then conducted to examine item comprehension and contextual suitability. For the relational job crafting scale, test-retest reliability was examined in this subsample after a two-week interval, yielding an acceptable test-retest coefficient of.82.

To rigorously validate the final measurement model, we randomly split the total sample (*N* = 642) into two subsamples: Subsample A (*n* = 321) for Exploratory Factor Analysis (EFA) and Subsample B (*n* = 321) for Confirmatory Factor Analysis (CFA). During the EFA phase, 20 items from the initial 56-item pool were deleted. Crucially, to preserve content validity and avoid overfitting the scales to the current sample, item deletion was driven by both statistical criteria and strict theoretical evaluation. While low factor loadings (*<.*50) or high cross-loadings flagged items for review, an item was only removed if the remaining indicators adequately captured the core conceptual domain. For instance, generic items that overlapped heavily with ordinary university life were removed to preserve the distinct vocational and contextual focus required for police cadets. To ensure absolute transparency, the complete list of all 56 original items—alongside the specific statistical and theoretical rationale for each deletion—is explicitly provided in the **Appendix** (**Tables A1, A2**).

To further verify that this item reduction did not bias the structural findings, a sensitivity analysis was conducted. We estimated the final structural equation model using all original 56 items and compared the results to the refined 36-item model. The direction, magnitude, and statistical significance of all primary structural paths remained highly consistent across both versions (e.g., the standardized path from reality shock to mental resilience was *β* = −.22 in the 56-item model versus *β* = −.23 in the 36-item model). Thus, a robust final set of 36 items was retained for the CFA and structural equation models to ensure measurement parsimony and clarity. There were 3 reverse-coded items. Construct scores were calculated by averaging the retained items after appropriately reversing the negatively worded items.

### 2.3 Measures

The final structural models were based on the 36 retained items.

#### 2.3.1 Positive psychological traits

Positive psychological traits were measured with an 8-item scale assessing optimism, hope, self-efficacy, perseverance, and positive coping orientation. Items were adapted from prior research on psychological capital and positive psychological resources (20, 21, 33). The wording was adjusted to reflect study and training life in police education. Sample items included “I can usually find a way to deal with difficulties in study or training” and “Even when training is stressful, I believe I can improve the situation”. Participants responded on a five-point scale from 1, strongly disagree, to 5, strongly agree. Higher scores indicated stronger positive psychological traits.

#### 2.3.2 Reality shock

Reality shock was measured with a 5-item scale adapted to the police education context. The items assessed the perceived gap between cadets’ prior image of police work and their current experience of police training. The scale was informed by research on work role transition, organizational entry, and socialization (9–12). Sample items included “Police training is more different from what I imagined than I expected” and “Some experiences in police education have made me reconsider my original view of this occupation”.

#### 2.3.3 Occupational calling

Occupational calling was assessed with a 6-item scale measuring perceived meaning, social value, and personal commitment related to future police work. The scale was adapted from calling research (22–24). Sample items included “I feel that police work is connected with an important purpose in my life” and “Becoming a police officer would allow me to contribute to society in a meaningful way”.

#### 2.3.4 Identity work support

Identity work support was measured with a 6-item scale developed based on identity work, professional identity, and organizational identification literature (26–29). The scale assessed whether cadets perceived support for exploring and constructing a police-related identity. Sample items included “In my training environment, I can discuss confusion about becoming a police officer” and “People around me help me understand what kind of police officer I want to become”.

#### 2.3.5 Relational job crafting

Relational job crafting was measured with a 5-item scale adapted from job crafting research (30–32, 34). Items assessed cadets’ proactive efforts to build, adjust, or use interpersonal relationships that helped them adapt to police training. Sample items included “I actively seek advice from people who understand police work” and “I try to build cooperative relationships that help me handle training demands”.

#### 2.3.6 Mental resilience

Mental resilience was measured with a 6-item scale assessing emotional recovery, adaptive coping, persistence, and psychological flexibility under stress. Items were informed by resilience measurement and developmental resilience literature (5, 14–16). Sample items included “I can recover after setbacks in study or training” and “When I face pressure, I can adjust myself and continue doing what needs to be done”.

#### 2.3.7 Control variables

Gender, age, academic year, only-child status, family residence, and whether the participant had a close family member with police experience were included as control variables. These variables were selected because they may be related to occupational expectations, access to support, developmental stage, and adaptation to police education.

### 2.4 Data analysis

Data were analyzed using SPSS 27.0 and Mplus 8.3. As described, Subsample A (*n* = 321) was used for EFA and Subsample B (*n* = 321) for CFA to validate the 36-item model. The structural mediation models were tested on the full sample (*N* = 642).

Regarding construct dimensionality, we carefully considered the theoretical facets of each construct. Although concepts like Positive Psychological Traits (e.g., optimism, hope, resilience) and Reality Shock encompass multiple facets theoretically, we modeled them as unidimensional reflective constructs at the scale level. This specification was chosen because our study focuses on the overarching latent traits (i.e., the general magnitude of psychological resources or overall environmental risk) rather than the distinct effects of specific sub-dimensions. The high internal consistency and strong factor loadings in the current sample supported this specification.

To provide comprehensive reliability and validity evidence, we calculated Cronbach’s *α*, McDonald’s *ω*, Composite Reliability (CR), and Average Variance Extracted (AVE) alongside their 95% confidence intervals. Discriminant validity was further verified using the Heterotrait-Monotrait ratio (HTMT) of correlations, adopting a conservative cutoff of *<.*85. Detailed HTMT confidence intervals are reported in the **Appendix**.

To test Hypotheses 3-7, we evaluated three competing structural models using the full sample. To accurately account for the degrees of freedom (df), the models were constructed as a strict nested sequence (a detailed mathematical nesting matrix listing all freely estimated and fixed parameters is provided in **Table A4** in the **Appendix**): (1) Model 1: Parallel Mediation Model (df = 602): Paths from the predictor to the mediators, and mediators to the outcome, were freely estimated. Crucially, paths *between* the three mediators were explicitly fixed to zero. (2) Model 2: Combined Parallel-Sequential Model (df = 600): Nested within Model 1. This model explicitly freed exactly two sequential paths between mediators (Calling → Identity, and Identity → Crafting). By adding these two freely estimated parameters, the degrees of freedom precisely dropped by 2 (602 - 2 = 600). (3) Model 3: Full Combined Model (df = 597): Nested within Model 2. This model additionally freed 3 paths to account for environmental risk (Reality Shock → Calling, Reality Shock → Identity, and Reality Shock → Crafting). Adding these 3 parameters reduced the df by exactly 3 compared to the previous model (600 - 3 = 597).

Since these models are nested, formal *χ*^2^ difference tests (Δ*χ*^2^) were used alongside AIC, BIC, and sample-size-adjusted BIC (ssaBIC) to identify the best-fitting model. Because the data were cross-sectional, we also explicitly tested reverse-directional alternative models to empirically evaluate the theoretical sequence. Indirect associations were tested with 5,000 bootstrap samples.

Finally, multi-group SEM was conducted to examine measurement and structural invariance. The grouping variable was academic year, comprising first-year (*n* = 218), second-year (*n* = 207), and thirdyear (*n* = 217) cadets. The models were estimated using Maximum Likelihood with robust standard errors (MLR). Nested constraints were sequentially applied to test configural invariance (no equality constraints), metric invariance (equal factor loadings), scalar invariance (equal item intercepts), and residual invariance (equal item residual variances). Based on recommended criteria, invariance was considered acceptable when the change in CFI (ΔCFI) was ≥−.010 and the change in RMSEA (ΔRMSEA) was ≤.015.

### 2.5 Ethical considerations

The study followed the principles of voluntary participation, informed consent, anonymity, and minimal risk. The research protocol was reviewed and approved by the Ethics Committee of Jiangsu Police Institute under approval number JSPI-ETH-2026-047. Because the participants were adolescent cadets and some were younger than 18 years old, the study procedure followed institutional requirements for research involving minors in educational settings. Participation was arranged only after permission had been obtained from the participating institutions. Cadets were informed that they could decline or withdraw without any negative consequence. For participants younger than 18 years old, guardian consent or institutionally approved parental consent procedures were obtained according to local ethical requirements.

## 3 Results

### 3.1 Descriptive statistics and reliability

**Table 1** presents the demographic characteristics of the adolescent police cadets. The final sample included 642 participants aged 16 to 20 years. **Table 2** presents means, standard deviations, and Pearson correlations among the variables. The correlation coefficients were moderate in size, suggesting that the constructs were related but not redundant.

**Table 2:** Means, SDs, and Pearson correlations.

Variable	M	SD	1	2	3	4	5	6
1. PPT	3.69	0.62	1					
2. Reality Shock	2.91	0.73	−.26^∗∗^	1				
3. Occ. Calling	3.63	0.71	.47^∗∗^	−.31^∗∗^	1			
4. Identity Support	3.48	0.68	.43^∗∗^	−.21^∗∗^	.46^∗∗^	1		
5. Relational Crafting	3.55	0.65	.45^∗∗^	−.18^∗∗^	.42^∗∗^	.45^∗∗^	1	
6. Mental Resilience	3.71	0.64	.57^∗∗^	−.36^∗∗^	.48^∗∗^	.44^∗∗^	.50^∗∗^	1

**Table 3** presents the advanced psychometric properties for the retained 36 items. All constructs exhibited excellent internal consistency, with Cronbach’s *α* and McDonald’s *ω* well above the acceptable thresholds (*ω* range:.86 to.93). Convergent validity was strongly supported, with Composite Reliability (CR) *>.*86 and Average Variance Extracted (AVE) *>.*54 for all scales.

**Table 3:** Reliability and convergent validity metrics for the retained scales (36 items).

Construct (No. of items)	*α*	*ω* [95% CI]	CR [95% CI]	AVE [95% CI]
1. Pos. Psych Traits (8)	.90	.91 [.88,.92]	.91 [.89,.93]	.58 [.55,.62]
2. Reality Shock (5)	.87	.87 [.85,.89]	.88 [.86,.90]	.61 [.58,.65]
3. Occupational Calling (6)	.91	.91 [.89,.93]	.92 [.90,.94]	.66 [.63,.70]
4. Identity Work Support (6)	.90	.90 [.88,.92]	.90 [.89,.92]	.62 [.59,.66]
5. Relational Job Crafting (5)	.88	.88 [.86,.90]	.89 [.87,.91]	.57 [.54,.61]
6. Mental Resilience (6)	.92	.92 [.90,.94]	.92 [.91,.94]	.63 [.60,.67]

CI, Confidence Interval.

Discriminant validity was established via the HTMT matrix (**Table 4**). All HTMT values were below the strict 0.85 threshold, confirming that the constructs are empirically distinct. Additional criterion-related validity evidence for the relational job crafting scale is detailed in **Table 5**.

**Table 4:** Heterotrait-Monotrait ratio (HTMT) matrix for discriminant validity.

Variable	1	2	3	4	5	6
1. PPT	–					
2. Reality Shock	.32 [.21,.43]	–				
3. Occ. Calling	.52 [.41,.61]	.35 [.24,.45]	–			
4. Identity Support	.48 [.38,.59]	.25 [.15,.36]	.51 [.40,.63]	–		
5. Relational Crafting	.50 [.40,.61]	.21 [.11,.32]	.47 [.35,.58]	.50 [.39,.62]	–	
6. Mental Resilience	.62 [.51,.73]	.40 [.29,.52]	.53 [.42,.64]	.49 [.38,.59]	.56 [.45,.67]	–

All HTMT values and their upper limits are below the strict 0.85 threshold.

**Table 5:** Criterion-related validity evidence for relational job crafting.

Criterion variable	r	95% CI lower	95% CI upper
Occupational calling	.42	.35	.48
Identity work support	.45	.38	.51
Mental resilience	.50	.44	.56
Positive psychological traits	.45	.38	.51
Reality shock	-.18	-.25	-.10

### 3.2 Measurement model validation and common method bias

Confirmatory factor analysis was conducted on Subsample B (*n* = 321) to examine the distinctiveness of the six constructs based on the retained 36 items. The hypothesized six-factor model showed excellent fit, *χ*^2^ = 971.48, df = 579, CFI = .936, TLI = .929, RMSEA = .046, SRMR = .045. Standardized factor loadings were significant and ranged from.62 to.87.

Several alternative models were tested for comparison. A five-factor model combining occupational calling and identity work support showed weaker fit, *χ*^2^ = 1846.21, df = 584, CFI = .896. A four-factor model combining occupational calling, identity work support, and relational job crafting into one resource factor also showed poorer fit, *χ*^2^ = 2234.66, df = 588, CFI = .864. A one-factor model fit the data poorly, *χ*^2^ = 4968.35, df = 594, CFI = .639, RMSEA = .107. The exact difference in degrees of freedom between the one-factor model (df = 594) and the six-factor model (df = 579) perfectly reflects the 15 freely estimated covariances among the six latent factors, supporting the empirical distinction among the six study variables.

Because all main variables were measured through self-report questionnaires, common method bias was considered. Harman’s single-factor test showed that the first unrotated factor explained 32.6 percent of the total variance. A common latent factor model did not produce a substantial improvement over the six-factor measurement model. These results suggest that one general method factor was unlikely to explain the overall pattern of findings.

### 3.3 Competing structural mediation and alternative directional models

We tested the structural paths using the full sample (*N* = 642). **Table 6** presents the formal nested comparisons. The *χ*^2^ difference tests revealed that the addition of the sequential paths in Model 2 did not significantly improve global model fit over the Parallel Model 1 (Δ*χ*^2^(2) = 2.81, *n.s.*). However, freeing the paths from reality shock to the three mediators in Model 3 (Full Combined Model) yielded a significant improvement over Model 2 (Δ*χ*^2^(3) = 9.27, *p <.*05). The AIC also favored the Full Combined Model. Although the global fit did not significantly jump strictly due to the sequential paths, the specific sequential indirect effect was theoretically hypothesized and subsequently tested via bootstrap analysis. Therefore, Model 3 was retained as the final structural model to comprehensively interpret all hypothesized parallel and sequential associations alongside environmental risks.

**Table 6:** Competing nested structural models comparison.

Model	*χ*2	df	CFI	RMSEA	AIC	BIC	ssaBIC	Δ*χ*^2^(Δ*df*)
1. Parallel Mediation	1441.73	602	.931	.047	45218.36	45450.12	45280.45	–
2. Combined Par-Seq	1438.92	600	.932	.047	45216.01	45456.23	45281.33	M1 vs M2: 2.81
3. Full Combined Model	1429.65*	597*	.934	.047	45210.44	45468.10	45283.67	M2 vs M3: 9.27

The Full Combined Model (Model 3) is the retained final model. ^∗^*p <.*05.

Because the data are cross-sectional and temporal causality cannot be definitively proven, we evaluated two theoretically plausible alternative structural models to cross-validate our proposed sequence. *Alternative Model A (Reverse Sequence)* tested whether relational job crafting predicted identity work support, which in turn predicted occupational calling (*χ*^2^ = 1485.42, df = 597, CFI = .921, RMSEA = .051, AIC = 45280.12). *Alternative Model B (Reverse Causality)* tested a structure where mental resilience acted as the primary antecedent predicting the mediators and positive psychological traits (*χ*^2^ = 1502.18, df = 597, CFI = .918, RMSEA = .054, AIC = 45311.65). Both alternative directional models yielded poorer fit indices and higher information criteria compared to our hypothesized Full Combined Model (AIC = 45210.44). This empirical comparison supports the theoretical rationale for our proposed ordered pathways, though causal conclusions remain strictly restricted.

Among the control variables (included in the final structural model), academic year showed a small positive association with reality shock, indicating that more advanced cadets reported slightly stronger reality shock. Gender, age, only-child status, family residence, and family police experience were not consistently associated with mental resilience after the main variables were included.

### 3.4 Path coefficients and indirect associations

Standardized path coefficients from the Full Combined Model are presented in **Table 7**. Reality shock was negatively associated with mental resilience (*β* = −.23, *p <.*001), supporting Hypothesis 1. Additionally, reality shock was negatively associated with occupational calling (*β* = −.19, *p <.*001), identity work support (*β* = −.14, *p <.*001), and relational job crafting (*β* = −.12, *p <.*01), indicating that environmental risk is linked to the depletion of these developmental resources. Positive psychological traits were positively associated with mental resilience (*β* = .27, *p <.*001), supporting Hypothesis 2.

**Table 7:** Standardized path coefficients (derived from the full combined model).

Path	Beta	SE	p
Reality shock → Mental resilience	-.23	.04	*<.*001
Reality shock → Occupational calling	-.19	.04	*<.*001
Reality shock → Identity work support	-.14	.04	*<.*001
Reality shock → Relational job crafting	-.12	.04	*<.*01
PPT → Mental resilience	.27	.05	*<.*001
PPT → Occupational calling	.47	.04	*<.*001
PPT → Identity work support	.40	.04	*<.*001
PPT → Relational job crafting	.44	.04	*<.*001
Occupational calling → Identity work support	.36	.04	*<.*001
Occupational calling → Mental resilience	.18	.04	*<.*001
Identity work support → Relational job crafting	.33	.05	*<.*001
Identity work support → Mental resilience	.17	.05	*<.*001
Relational job crafting → Mental resilience	.22	.04	*<.*001

Bootstrap analyses (5,000 samples) confirmed the indirect pathways (**Table 8**). The full sequential pathway linking positive psychological traits to mental resilience through occupational calling, identity work support, and relational job crafting was significant (*estimate* = .012, 95% CI [.005*,.*026]). This supports Hypothesis 7 at the specific indirect effect level. However, its effect size (.012) is notably smaller than the parallel pathways through calling alone (.085), identity support alone (.068), and relational crafting alone (.097). These findings suggest that while the sequential pathway is structurally viable, the mediating mechanisms operate primarily as parallel pathways with a supplementary sequential dynamic.

**Table 8:** Bootstrap indirect effects (derived from the full combined model).

Indirect pathway	Estimate	SE	95% CI
Sequential: PPT → Call → Identity → Craft → Resilience	.012	.005	[.005,.026]
Parallel 1: PPT → Call → Resilience	.085	.018	[.051,.122]
Parallel 2: PPT → Identity → Resilience	.068	.017	[.035,.108]
Parallel 3: PPT → Craft → Resilience	.097	.022	[.061,.142]

### 3.5 Academic-year multi-group SEM

Multi-group SEM was conducted to examine measurement and structural invariance across academic years. As shown in **Table 9**, the configural invariance model showed acceptable fit. The changes in fit indices for the metric (ΔCFI = +.002, ΔRMSEA = -.001), scalar (ΔCFI = -.001, ΔRMSEA = -.001), and residual (ΔCFI = -.004, ΔRMSEA = +.001) invariance models were well within the predefined acceptable criteria. These results confirmed full measurement equivalence across training stages, and no partial-invariance procedures were required.

**Table 9:** Measurement invariance across academic years.

Model	*χ*2	df	CFI	RMSEA	SRMR	ΔCFI	ΔRMSEA
Configural	2416.38	1737	.924	.043	.058	–	–
Metric	2468.21	1805	.926	.042	.061	+.002	-.001
Scalar	2531.74	1873	.925	.041	.064	-.001	-.001
Residual	2610.50	1909	.921	.042	.065	-.004	+.001

Grouping variable: Academic year (First year *n* = 218, Second year *n* = 207, Third year *n* = 217). Models estimated using MLR. ΔCFI and ΔRMSEA reflect changes relative to the preceding, less constrained model. Full strict measurement invariance was supported; no partial-invariance procedures were required.

After establishing measurement invariance, structural paths were compared across academic years (see **Table 10**). The negative association between reality shock and mental resilience appeared slightly stronger among third-year cadets than among first-year cadets. However, most structural paths were similar across academic years, and constraining the main structural paths to equality did not substantially worsen model fit. These results suggest that while academic year may be related to the level of reality shock, the broader pattern linking psychological resources, meaning, identity, relational crafting, and resilience is robust and generally stable.

**Table 10:** Selected structural paths across academic years.

Path	First year	Second year	Third year
Reality shock → Mental resilience	−.18^∗∗^	−.22^∗∗∗^	−.28^∗∗∗^
Positive psychological traits → Mental resilience	.29∗∗∗	.27∗∗∗	.24∗∗∗
Positive psychological traits → Occupational calling	.45∗∗∗	.48∗∗∗	.47∗∗∗
Positive psychological traits → Identity work support	.39∗∗∗	.41∗∗∗	.40∗∗∗
Positive psychological traits → Relational job crafting	.43∗∗∗	.44∗∗∗	.45∗∗∗
Occupational calling → Mental resilience	.17^∗∗^	.18^∗∗^	.19^∗∗^
Identity work support → Mental resilience	.16^∗^	.17^∗∗^	.18^∗∗^
Relational job crafting → Mental resilience	.22∗∗∗	.23∗∗∗	.24∗∗∗

## 4 Discussion

This study examined mental resilience among adolescent police cadets from an environmental risk and resilience perspective. The aim was not to establish causal conclusions, but to clarify how reality shock, positive psychological traits, occupational calling, identity work support, and relational job crafting were associated with resilience in a distinctive police academy context. The findings suggest that adolescent police cadets’ resilience is linked to both personal traits and the social meanings available in the training environment.

Reality shock was negatively associated with mental resilience. This result is consistent with the interpretation that the gap between imagined policing and experienced police training may function as a vocational environmental risk. Furthermore, the structural model indicated that reality shock was also negatively associated with occupational calling, identity work support, and relational job crafting. This suggests that the expectation-reality discrepancy does not merely act as a direct stressor; it may also erode the core developmental resources and meanings that cadets rely on to navigate academy life. The finding supports the need to distinguish police academy reality shock from general academic setback.

The police academy context gives reality shock several possible dimensions. Task-related shock may emerge from repetitive training and physical demands. Organizational shock may emerge from hierarchical discipline, collective schedules, and limited autonomy. Emotional shock may emerge from expectations of restraint and composure. Relational shock may emerge from peer comparison, command-based interaction, and group living. Occupational-ethical shock may emerge when cadets discover that police work is often routine, constrained, publicly scrutinized, and sometimes morally complex. These dimensions suggest that reality shock among police cadets is a multidimensional vocational experience rather than a simple response to school difficulty.

Positive psychological traits were positively associated with mental resilience. Cadets who reported stronger optimism, hope, self-efficacy, perseverance, and positive coping orientation also reported stronger adaptive capacity. This result is consistent with prior work on psychological capital and positive psychological resources (20, 21, 33). However, the mediation findings caution against an overly individualistic interpretation. Positive psychological traits were linked to resilience partly through occupational calling, identity work support, and relational job crafting. This pattern suggests that personal strengths may be more closely associated with resilience when cadets can connect them with meaningful occupational narratives, identity resources, and supportive relationships.

Occupational calling was one meaning-based pathway. Among adolescent police cadets, calling may help cadets interpret training demands as connected with public service rather than as meaningless burden. However, calling should not be understood as simple enthusiasm. A fragile calling may depend on heroic or idealized images of policing and may weaken when cadets encounter routine work or institutional constraints. A more mature calling includes both value and realism. Police education may therefore support cadets not by preserving unrealistic idealism, but by helping them develop realistic commitment.

Identity work support was another pathway. This finding is especially relevant because the sample consisted of adolescents and late adolescents. Police education accelerates identity construction by asking cadets to imagine themselves as future law enforcement professionals. This role contains tensions between authority and service, discipline and discretion, emotional control and empathy, organizational loyalty and legal responsibility. When cadets perceive support for discussing these tensions, identity confusion may become a developmental task rather than a private burden.

Relational job crafting was also positively linked to resilience. Cadets rarely experience academy life as isolated individuals. They live, train, compete, and recover in groups. Relationships with classmates, senior cadets, instructors, counselors, and field mentors shape how the same training demand is interpreted. Relational job crafting gives cadets a form of agency in an environment where many formal rules cannot be changed by students. By seeking advice, building trust, and developing cooperative ties, cadets may gain access to information, emotional support, and practical feedback.

The competing sequential mediation and alternative directional tests clarified the architecture of this process. The data statistically rejected reverse-causality models and supported a combined parallelsequential pattern. While parallel pathways carried the largest indirect associations, a significant supplementary sequential pathway emerged: greater psychological traits were linked to stronger calling, stronger calling was linked to greater identity support, identity support was linked to more active relational crafting, and relational crafting was tied to mental resilience. This suggests that while cadets draw on these resources simultaneously, there is also a logical developmental progression where motivational meaning serves as the bedrock upon which professional identity is constructed, subsequently empowering proactive relational behaviors.

### 4.1 Chinese police academy context

The Chinese police academy context is important for interpreting these findings. Compared with many European and North American police training systems, Chinese police education often begins earlier, is embedded in higher education, and involves collective living, enclosed or semi-enclosed management, regularized discipline, and strong occupational identity formation before formal employment. These features make Chinese police cadets different from both ordinary adolescents and adult police recruits.

This context may have both protective and risk-related implications. On the one hand, collective routines, clear rules, and shared occupational goals may provide belonging, predictability, and purpose. On the other hand, limited autonomy, close peer comparison, early professional expectations, and strong institutional discipline may make expectation-reality discrepancies more salient. Therefore, the findings should not be assumed to generalize directly to police training systems in other countries. Cross-cultural research is needed to examine whether similar mechanisms appear in police education systems with different admission ages, training structures, occupational cultures, and institutional expectations.

### 4.2 Theoretical contributions

This study contributes to adolescent mental health research by bringing attention to a specialized educational environment that has rarely been placed within an environmental risk and resilience framework. Police academies are not ordinary schools, and adolescent police cadets are not simply future employees. Their setting combines schooling, discipline, collective life, occupational values, and early professional socialization. This combination makes the police academy a meaningful context for studying how institutional environments are linked to adolescent resilience.

The study also extends the concept of reality shock. Previous research has usually discussed reality shock in relation to organizational entry, work role transition, or early career adaptation (9–11). The present study suggests that reality shock may emerge before formal employment, especially in vocational education settings where occupational identity is formed early.

Another contribution concerns the process view of resilience. Resilience is sometimes described in everyday language as toughness. The present model provides a more ecological and developmental account. Positive psychological traits were associated with resilience, but part of this association passed through calling, identity work support, and relational job crafting. This view is consistent with ecological theories of resilience that emphasize person-environment interaction rather than isolated individual traits (6–8).

Finally, the study connects research on calling, identity work, and job crafting in a single adolescent training context. These literatures are often separated. Calling research focuses on meaning and purpose, identity work research focuses on self-construction, and job crafting research focuses on proactive changes in work boundaries (22, 28, 30). In police education, however, these processes overlap. A cadet’s sense of calling may depend on identity support. Identity development may depend on relationships. Relational agency may help sustain meaning.

### 4.3 Practical implications

Several practical implications follow from the findings. First, police education programs may benefit from managing occupational expectations more deliberately. Cadets often arrive with partial images of police work. Institutions do not need to remove idealism, but they may help cadets build realistic idealism through introductory courses, alumni conversations, supervised field observations, and guided discussions.

Second, resilience education should not be limited to messages about endurance. Cadets also need tools for interpreting stress, discussing confusion, and seeking support. Reflection journals, small group conversations, and structured debriefings after difficult training experiences may help turn pressure into learning rather than silent accumulation.

Third, occupational calling should be cultivated in a mature form. Police academies often emphasize service, sacrifice, and responsibility. These values are important, but they should not be presented in a way that leaves no room for doubt. A cadet may feel tired, disappointed, or uncertain and still care about public service.

Fourth, identity work support should be made visible in academy life. Cadets need spaces where they can ask what kind of police officer they want to become. These spaces can be built into mentoring systems, class meetings, counseling services, case discussions, and field practice debriefings.

Fifth, police academies may encourage relational job crafting. Because academy life is highly structured, cadets may feel that adaptation is imposed on them. Relational job crafting provides a practical form of agency through advice seeking, cooperative learning, peer support, and mentor relationships.

### 4.4 Limitations and future research

Several limitations should be noted. First, the cross-sectional design strictly precludes causal conclusions. Although our proposed structural ordering was built upon developmental and organizational theory—and was empirically supported over alternative reverse-sequence models—it remains possible that these variables are reciprocally related. For example, while calling may facilitate identity support, more resilient cadets might also naturally report stronger calling and engage in more relational job crafting. Future longitudinal research is heavily needed to establish the precise temporal ordering of reality shock and resilience across training stages. A useful design would follow cadets from admission through field practice and early employment.

Second, all main variables were measured through self-report questionnaires. This approach is appropriate for subjective experiences such as reality shock, calling, and perceived support, but it may introduce common method bias and social desirability. This issue is especially relevant in police education, where cadets may be cautious when reporting negative experiences. Future studies could include interviews, diary methods, peer reports, instructor ratings, or behavioral indicators of adjustment.

Third, the sample came from three police education institutions in China. Police education systems differ across regions and countries. Admission age, training intensity, academy culture, employment pathways, and public attitudes toward policing may shape cadets’ experiences. Comparative studies across institutions and cultural contexts would help clarify which findings are context-specific and which are more general.

Fourth, although additional psychometric evidence was provided for the adapted relational job crafting scale, further validation remains needed. Future research should examine the scale in independent samples, test its predictive validity in longitudinal designs, and compare it with behavioral indicators of relational adaptation in police academy settings.

Fifth, this study treated reality shock as a general construct in the main statistical model. In practice, reality shock may include task-related, organizational, emotional, relational, and occupational-ethical dimensions. Future studies should develop and validate a multidimensional police academy reality shock scale.

Finally, although academic-year differences were explored through multi-group SEM, longitudinal designs are needed to examine developmental change. Following cadets from admission through field practice and early employment would provide stronger evidence about how reality shock, calling, identity work, relational crafting, and resilience unfold over time.

## 5 Conclusion

Adolescent police cadets develop in a distinctive environment where schooling, discipline, collective life, and occupational socialization overlap. In this setting, reality shock was negatively associated with mental resilience and may represent a vocational environmental risk when cadets experience a mismatch between idealized images of policing and the realities of training. Positive psychological traits were positively associated with resilience, and this association was partly linked to occupational calling, identity work support, and relational job crafting. These findings suggest that resilience in adolescent police cadets should not be reduced to individual toughness. It is also related to whether cadets can find meaning in police work, receive support while forming a professional identity, and build constructive relationships within the training environment. Because the study used primarily cross-sectional self-report data, the findings should be interpreted as associations rather than causal effects.

## Data Availability

The datasets presented in this article are not readily available because they contain information that could compromise the privacy of the adolescent participants, and the study was conducted with the assurance of strict anonymity. Under the terms of the ethical approval granted by the Ethics Committee of Jiangsu Police Institute (approval number: JSPI-ETH-2026-047), the data cannot be made publicly accessible. Requests to access the de-identified datasets should be directed to the corresponding author, Hongping Lin (adrs2417@outlook.com), who will assess each request for research integrity and compliance with ethical standards.
