# Supplementary material for: Reality shock and mental resilience among adolescent police cadets: meaning, identity, and relational pathways in a Chinese police academy context
Source: Front Psychiatry. 2026 Oct 5;17:1921313. doi: 10.3389/fpsyt.2026.1921313 (PMC13678299; doi:10.3389/fpsyt.2026.1921313)
Supplement: Supplementary file 1 [file SupplementaryFile1.pdf]

Appendix: Comprehensive item lists and structural specifications

A1. Complete 56-item pool and theoretical deletion rationale

To guarantee full transparency and address content validity, Tables A1 and A2 list the entirety of the original 56 items generated during scale adaptation. They detail the exact theoretical reasons why 20 items were removed during the EFA stage. Item deletion was rigorously governed by a rule to strictly preserve vocational and contextual specificity (e.g., removing items that measured behavioral compliance rather than psychological traits, or general academic stress rather than police reality shock).

TABLE 11 Complete item pool, translations, and deletion rationale (part 1).

Item	Original Wording	Adapted Chinese Wording	Status & Strict Theoretical Rationale
**Positive Psychological Traits (Retained = 8, Deleted = 2)**			
PPT1	I find ways to deal with difficulties.	我通常能找到办法解决学习中的困难。	Retained

。

Item	Original Wording	Adapted Chinese Wording	Status & Strict Theoretical Rationale
PPT2	I feel confident analyzing problems.	我有信心分析复杂的问题。	Deleted: Overly cognitive, overlaps with academic intelligence rather than psychological resilience.
PPT3	I recover quickly from setbacks.	遇到挫折时，我能迅速恢复状态。	Retained
PPT4	I believe I can improve stressful situations.	即使压力大，我相信能改善状况。	Retained
PPT5	I can adapt to unexpected demands.	我能适应突如其来的要求。	Retained
PPT6	I usually look on the bright side.	我通常能看到事物积极的一面。	Retained

Item	Original Wording	Adapted Chinese Wording	Status & Strict Theoretical Rationale
PPT7	I trust my own training skills.	我对自己的训练技能有信心。	Deleted: Measures specific physical self-efficacy, not generalized psychological traits.
PPT8	I persevere even when exhausted.	即使疲惫，我也能坚持下去。	Retained
PPT9	I set goals for my own growth.	我会为自己的成长设定目标。	Retained
PPT10	I approach challenges with hope.	我以充满希望的态度面对挑战。	Retained
**Reality Shock (Retained = 5, Deleted = 5)**			

Item	Original Wording	Adapted Chinese Wording	Status & Strict Theoretical Rationale
RS1	Training differs from what I expected.	警校训练和我预想的差异很大。	Retained
RS2	Strict hierarchy was surprising to me.	严格的等级制度让我感到惊讶。	Retained
RS3	Routine tasks feel meaningless.	有时例行任务让我感觉毫无意义。	Deleted: Confounded with burnout/cynicism rather than expectation-mismatch.
RS4	Experiences made me reconsider my view.	某些经历让我重新思考了对警察的看法。	Retained
RS5	Emotional restraint is tougher than expected.	所需的情感控制比我想象的更难。	Retained

Item	Original Wording	Adapted Chinese Wording	Status & Strict Theoretical Rationale
RS6	The physical pain is unbearable.	身体上的疲劳难以忍受。	Deleted: Focuses entirely on physical stamina, losing the psychological element of reality shock.
RS7	The curriculum is difficult.	这里的文化课程非常难。	Deleted: Describes generic academic stress found in any university, lacks police context.
RS8	Instructors are less supportive than imagined.	教官比我想象的要严厉。	Retained
RS9	I worry about failing exams.	我常担心自己考试不及格。	Deleted: Generic academic anxiety.
RS10	The food and dorms are disappointing.	食宿条件让我感到失望。	Deleted: Captures lifestyle dissatisfaction, irrelevant to vocational role transition.
**Occupational Calling**			

Item	Original Wording	Adapted Chinese Wording	Status & Strict Theoretical Rationale
**(Retained = 6, Deleted = 4)**			
OC1	Connected with an important purpose.	警察工作与我生命中重要的目标相连。	Retained
OC2	I feel destined to do this work.	我觉得自己注定要做这份工作。	Retained
OC3	Willing to sacrifice personal time.	愿意为职业牺牲个人时间。	Deleted: Confounded with workaholism or organizational exploitation.
OC4	Contribute to society meaningfully.	成为警察能让我为社会做出贡献。	Retained
OC5	It is a highly respected job.	警察是一个受人尊	Deleted: Measures perceived social prestige, not

Item	Original Wording	Adapted Chinese Wording	Status & Strict Theoretical Rationale
		敬的职业。	internalized personal calling.
OC6	The work aligns with my values.	这份工作符合我的核心价值观。	Retained
OC7	I enjoy the uniform.	我喜欢穿警服带来的感觉。	Deleted: Focuses on superficial aesthetic rewards rather than deep purpose.
OC8	It provides financial stability.	这份工作能提供稳定的收入。	Deleted: Extrinsic motivation, opposite of the theoretical construct of Calling.
OC9	I feel driven to protect others.	我内心有强烈的保护他人的动力。	Retained
OC10	I am passionate about law enforcement.	我对执法工作充满热情。	Retained

(Continued)

TABLE 11 Continued

Item	Original Wording	Adapted Chinese Wording	Status & Strict Theoretical Rationale
**Identity Work Support (Retained = 6, Deleted = 4)**			
ID1	Can discuss confusion about becoming police.	我可以与他人讨论成为警察的困惑。	Retained
ID2	Encouraged to express true self.	环境鼓励我表达真实的自我。	Deleted: Directly contradicts police academy culture emphasizing discipline over individual expression.
ID3	Instructors provide constructive feedback.	教官会提供建设性的反馈。	Deleted: Measures specific instructional quality rather than broader identity support.
ID4	Help me understand the	周围人帮我理解我	Retained

Item	Original Wording	Adapted Chinese Wording	Status & Strict Theoretical Rationale
	officer I want to be.	想成为怎样的警察。	
ID5	I am taught the history of policing.	课堂上教授了警察的历史。	Deleted: Measures curriculum content, not relational identity construction.
ID6	Mentors help me reconcile role conflicts.	导师帮我调和角色上的冲突感。	Retained
ID7	Peer discussions clarify my professional role.	同学讨论让我更清晰自己的职业角色。	Retained
ID8	I have role models to look up to.	这里有值得我学习的警察榜样。	Retained

Item	Original Wording	Adapted Chinese Wording	Status & Strict Theoretical Rationale
ID9	Academy rules are clearly written.	警校的规章制度写得很清楚。	Deleted: Measures administrative clarity, entirely missing identity exploration.
ID10	I feel safe exploring career doubts here.	在这里表达职业疑虑是安全的。	Retained
**Relational Job Crafting (Retained = 5, Deleted = 3)**			
JC1	Actively seek advice from people.	我积极向了解警察工作的人寻求建议。	Retained
JC2	Organize study groups.	主动组织学习小组。	Deleted: Confounded with basic academic study habits rather than relational coping.

Item	Original Wording	Adapted Chinese Wording	Status & Strict Theoretical Rationale
JC3	Avoid people who complain.	有意避开那些经常抱怨的人。	Deleted: Measures avoidance behavior, violating the proactive definition of job crafting.
JC4	Build cooperative relationships.	建立能帮我应对训练要求的合作关系。	Retained
JC5	I ask senior cadets for their experiences.	我经常向高年级师兄师姐请教经验。	Retained
JC6	I connect with mentors outside class.	课外我会主动联系导师交流。	Retained
JC7	I follow orders exactly as given.	我严格按照命令执行任务。	Deleted: Captures strict behavioral compliance, fundamentally opposite to proactive crafting.

Item	Original Wording	Adapted Chinese Wording	Status & Strict Theoretical Rationale
JC8	I try to inspire my peers.	我努力去激励我周围的同学。	Retained
**Mental Resilience (Retained = 6, Deleted = 2)**			
MR1	Recover after setbacks.	遭遇挫折后能迅速恢复。	Retained
MR2	Easily adapt to rule changes.	轻松适应规则的改变。	Deleted: Reflects organizational flexibility/obedience rather than psychological bounce-back.
MR3	Rarely lose temper.	很少发脾气或失控。	Deleted: Confounded with emotional suppression/control, separate from resilience.

Item	Original Wording	Adapted Chinese Wording	Status & Strict Theoretical Rationale
MR4	Adjust myself and continue under pressure.	面对压力能自我调整并继续。	Retained
MR5	I stay calm during emergencies.	突发情况下我能保持冷静。	Retained
MR6	I bounce back from critical feedback.	受到严厉批评后我能振作起来。	Retained
MR7	I maintain focus during fatigue.	极度疲劳时我也能保持专注。	Retained
MR8	Difficulties make me stronger.	困难只会让我变得更强大。	Retained

A2. Explicit path specifications for the competing nested models

To fully clarify the exact degrees of freedom (df = 602, 600, 597) associated with the competing structural models described in Section 2.4, Table A4 provides a strict

accounting matrix. This matrix explicitly lists the freely estimated versus fixed paths, detailing the structural nesting of the models (Model 1 ⊂ Model 2 ⊂ Model 3).

TABLE 13 Mathematical nesting matrix for the competing structural models.

Structural Path	Model 1 (Parallel, df = 602)	Model 2 (Par-Seq, df = 600)	Model 3 (Full Combined, df = 597)
PPT → Calling	Free	Free	Free
PPT → Identity	Free	Free	Free
PPT → Crafting	Free	Free	Free
PPT → Resilience	Free	Free	Free
Calling → Resilience	Free	Free	Free
Identity → Resilience	Free	Free	Free
Crafting → Resilience	Free	Free	Free
Reality Shock → Resilience	Free	Free	Free
**Nested Sequential Paths:**			
Calling → Identity	Fixed to 0	Free (-1 df)	Free

Structural Path	Model 1 (Parallel, df = 602)	Model 2 (Par-Seq, df = 600)	Model 3 (Full Combined, df = 597)
Identity → Crafting	Fixed to 0	Free (-1 df)	Free
**Nested Environmental Paths:**			
Reality Shock → Calling	Fixed to 0	Fixed to 0	Free (-1 df)
Reality Shock → Identity	Fixed to 0	Fixed to 0	Free (-1 df)
Reality Shock → Crafting	Fixed to 0	Fixed to 0	Free (-1 df)
Total Structural Parameters	8	10	13
Resulting Degrees of Freedom	602	600	597

Note. All three models also included identically freely estimated paths from the 6 control variables to mental resilience (omitted here for brevity). The sequential progression accounts for the exact drop in degrees of freedom (-2, then -3).

A3. Path Diagrams for the Competing Structural Models

As requested, the following figures visually depict the nesting structure of the three competing structural models detailed in Table A4. Solid lines indicate freely estimated structural paths. Omitted paths between latent variables indicate parameters explicitly fixed to zero.

Figure 1. Model 1: The Parallel Mediation Model (df = 602). Reality Shock and Positive Psychological Traits (PPT) predict Mental Resilience. PPT also predicts the three mediators, which in turn predict Mental Resilience. Crucially, all possible paths between the three mediators are fixed to zero, and paths from Reality Shock to the mediators are fixed to zero.

Figure 2. Model 2: The Combined Parallel-Sequential Model (df = 600). Nested within Model 1, this model explicitly frees exactly two sequential paths (Calling → Identity, and Identity → Crafting). By releasing these two parameters, the degrees of freedom drop by 2.

Figure 3. Model 3: The Full Combined Model (df = 597). Nested within Model 2, this model explicitly frees three additional paths to account for environmental risk (Reality Shock → Calling, Reality Shock → Identity, and Reality Shock → Crafting). By releasing these three parameters, the degrees of freedom drop by 3.
